# Susceptibility of the human retrovirus XMRV to antiretroviral inhibitors

**DOI:** 10.1186/1742-4690-7-70

**Published:** 2010-08-31

**Authors:** Robert A Smith, Geoffrey S Gottlieb, A Dusty Miller

**Affiliations:** 1 Department of Pathology, University of Washington, Seattle WA, USA; 2 Department of Medicine, University of Washington, Seattle WA, USA; 3 Human Biology Division, Fred Hutchinson Cancer Research Center, Seattle WA, USA

## Abstract

**Background:**

XMRV (xenotropic murine leukemia virus-related virus) is the first known example of an exogenous gammaretrovirus that can infect humans. A limited number of reports suggest that XMRV is intrinsically resistant to many of the antiretroviral drugs used to treat HIV-1 infection, but is sensitive to a small subset of these inhibitors. In the present study, we used a novel marker transfer assay to directly compare the antiviral drug sensitivities of XMRV and HIV-1 under identical conditions in the same host cell type.

**Results:**

We extend the findings of previous studies by showing that, in addition to AZT and tenofovir, XMRV and HIV-1 are equally sensitive to AZddA (3'-azido-2',3'-dideoxyadenosine), AZddG (3'-azido-2',3'-dideoxyguanosine) and adefovir. These results indicate that specific 3'-azido or acyclic nucleoside analog inhibitors of HIV-1 reverse transcriptase (RT) also block XMRV infection with comparable efficacy *in vitro*. Our data confirm that XMRV is highly resistant to the non-nucleoside RT inhibitors nevirapine and efavirenz and to inhibitors of HIV-1 protease. In addition, we show that the integrase inhibitors raltegravir and elvitegravir are active against XMRV, with EC_50 _values in the nanomolar range.

**Conclusions:**

Our analysis demonstrates that XMRV exhibits a distinct pattern of nucleoside analog susceptibility that correlates with the structure of the pseudosugar moiety and that XMRV is sensitive to a broader range of antiretroviral drugs than has previously been reported. We suggest that the divergent drug sensitivity profiles of XMRV and HIV-1 are partially explained by specific amino acid differences in their respective protease, RT and integrase sequences. Our data provide a basis for choosing specific antiretroviral drugs for clinical studies in XMRV-infected patients.

## Background

The genus *gammaretroviridae *includes several well-characterized exogenous retroviruses that cause leukemia, lymphoma and other diseases in their natural hosts [1]. Although gammaretroviruses have been isolated from several vertebrate species, until recently, the only evidence that these agents could infect humans was the strong sequence similarity between certain human endogenous retroviruses and gammaretroviruses from other mammalian species [2]. In 2006, Urisman and colleagues reported the discovery of novel gammaretroviral cDNA sequences in tumor samples from patients with prostate cancer [3]. Full-length viral clones derived from the patient tissues were shown to be genetically similar to xenotropic strains of murine leukemia virus (MLV), and thus, the novel retrovirus was named xenotropic MLV-related virus (XMRV).

Subsequent studies have provided compelling evidence that XMRV is indeed the first known example of an exogenous human gammaretrovirus. XMRV sequences have been identified in tumor samples from three additional cohorts of prostate cancer patients [4-6], in a prostate carcinoma cell line [7], and in secretions expressed from cancerous prostate tissues [8]. Virus produced from a full-length XMRV molecular clone can infect primary prostate cells in culture, as well as several immortalized cell lines [7-12], and gammaretrovirus-like particles have been identified in XMRV-infected cultures by electron microscopy [5,7]. Although XMRV lacks direct transforming activity, foci of transformed cells appear at low frequencies in XMRV-infected fibroblast cultures, suggesting that the virus is capable of promoting carcinogenesis via insertional activation of cellular oncogenes [13]. Importantly, the chromosomal locations of XMRV proviruses have been mapped in tissue samples from 9 different patients with prostate cancer, confirming that XMRV can integrate into the human genome *in vivo *[11,14].

Following the discovery of XMRV in prostate tumor tissues, a PCR-based survey identified XMRV DNA in peripheral blood mononuclear cells (PBMC) from 68 of 101 chronic fatigue syndrome (CFS) patients living in the United States, as well as 8 of 218 healthy controls [15]. Remarkably, co-culture experiments revealed the presence of infectious XMRV in activated PBMC and in cell-free plasma samples from PCR-positive CFS patients, suggesting that these individuals harbor significant levels of replication-competent XMRV in the periphery. Although other studies of CFS and prostate cancer patients living outside the United States have failed to detect XMRV [16-20], data showing that the virus can infect human cells *in vitro *[7-12] and *in vivo *[11,14] provide a solid rationale for identifying antiviral inhibitors that block XMRV replication.

A growing body of evidence suggests that XMRV is intrinsically resistant to many of the drugs used to treat HIV-1 infection, but is sensitive to a small subset of antiretroviral inhibitors. In an initial analysis of XMRV drug susceptibility, treatment of immortalized prostate cells with 30 nM AZT inhibited XMRV infection by a factor of 25-fold; equivalent concentrations of other antiretroviral drugs had no effect on XMRV infection [21]. A subsequent study in cultured cells found that XMRV and HIV-1 exhibit comparable sensitivities to AZT, tenofovir disoproxil fumarate (TDF), and raltegravir suggesting that these drugs are relatively potent inhibitors of XMRV replication [22]. Finally, Singh *et al. *reported that AZT, TDF, raltegravir and the integrase inhibitor L-870812 inhibit XMRV infection at nanomolar concentrations in culture [23]. Although drug susceptibility data for HIV-1 were also presented, direct comparisons between XMRV and HIV-1 could not be made due to the differing cell types used to assay these viruses (*i.e*., immortalized breast and prostate cancer cells for XMRV versus primary blood lymphocytes for HIV-1) [23].

In the present study, we examined the ability of specific reverse transcriptase (RT), protease, and integrase inhibitors to block XMRV infection in culture by directly comparing the antiretroviral drug susceptibilities of XMRV and HIV-1 in the same host cell type. Our use of the same target cells for both viruses was particularly critical for assessing nucleoside RT inhibitor (NRTI) susceptibility, since the antiviral activity of these drugs varies widely in different host cell environments [23,24]. We also used conditions that restricted viral replication to a single cycle of infection to ensure that our drug susceptibility measurements were not influenced by differences in the relative replication rates of HIV-1 and XMRV. As in previous reports, we found that XMRV is intrinsically resistant to nevirapine, efavirenz, foscarnet, and all FDA-approved inhibitors of HIV-1 protease. However, our data also show that in addition to AZT and tenofovir, XMRV and HIV-1 are comparably sensitive to other structurally-related NRTIs. These findings reveal a distinct pattern of NRTI sensitivity in XMRV that correlates with the structure of the pseudosugar moiety. We also demonstrate that the integrase inhibitor elvitegravir suppresses XMRV infection with an EC_50 _similar to that of AZT, whereas raltegravir is the most potent anti-XMRV agent of all the inhibitors tested. These data suggest that the inhibitor-binding surfaces of HIV-1 and XMRV integrase share similar topologies despite numerous differences in their respective amino acid sequences. Collectively, our study reveals important features of the inhibitor specificities of XMRV RT and integrase and expands the number of antiretroviral drugs that are active against XMRV in culture.

## Results

### Comparison of HIV-1 and XMRV drug susceptibilities

We used a previously-described marker rescue assay [7,25] in conjunction with a Tat-inducible, β-gal-expressing HeLa cell line (MAGIC-5A) [26] to quantify the susceptibility of XMRV to antiretroviral inhibitors. Our XMRV stocks were derived from two independently-isolated strains of the virus: XMRV_VP62 _and XMRV_22Rv1_. XMRV_VP62 _was produced from a full-length molecular clone (pVP62) that was previously constructed by joining two overlapping cDNA fragments amplified from prostate tumor tissues [3,11]. For our experiments, high-titer XMRV_VP62 _stocks were generated by transfecting pVP62 into LNCaP prostate cancer cells [11]. XMRV_22Rv1 _was originally discovered in a prostate carcinoma cell line (22Rv1) that had been grown by xenotransplantation in nude mice [7,27]. 22Rv1 cells contain multiple integrated copies of the XMRV genome and release high titers of infectious XMRV into the culture supernatant [7].

**Figure 1:**
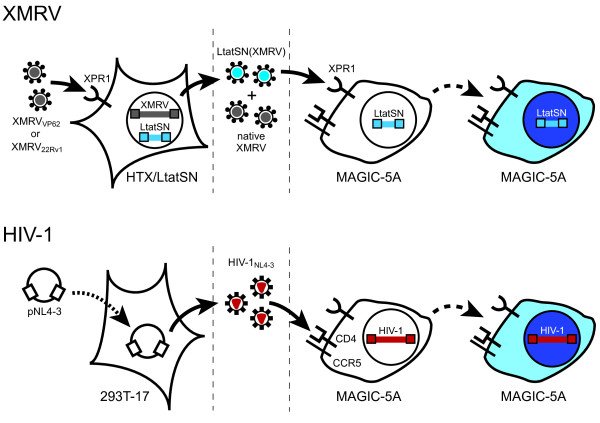
**Drug susceptibility assays for XMRV and HIV-1**. For XMRV, HTX/LtatSN cells were infected (solid arrows) with XMRV_22Rv1 _or XMRV_VP62_, resulting in the release of native XMRV (gray virions) as well as XMRV-pseudotyped virions that contain LtatSN RNA (LtatSN(XMRV); blue virions). Infection of MAGIC-5A cells with XMRV+LtatSN results in transfer of the HIV-1 *tat *marker gene, thereby inducing β-gal expression through Tat-dependent transactivation of an upstream HIV-1 LTR promoter. β-gal^+ ^(blue) cells are detected by staining the MAGIC-5A monolayers with X-gal (dashed arrows). Entry of XMRV into HTX/LtatSN and MAGIC-5A cells is mediated by the endogenously-expressed xenotropic and polytropic retrovirus receptor 1 (XPR1). For HIV-1, virus stocks were produced by transient transfection (dotted arrow) of 293T/17 cells with pNL4-3. As with XMRV+LtatSN, infection of MAGIC-5A cells with HIV-1_NL4-3 _(red virions) results in Tat expression and β-gal^+ ^focus formation. MAGIC-5A cells were previously engineered to express the CD4 receptor and CCR5 coreceptor for HIV-1 entry; these cells also express the endogenous CXCR4 coreceptor [26]. Dashed vertical lines indicate the stages at which protease inhibitors (left) and reverse transcriptase or integrase inhibitors (right) were added to the culture supernatants.

To generate viruses for drug susceptibility testing, HTX human fibrosarcoma cells were transduced with an MLV vector encoding HIV-1 *tat *(LtatSN) and were subsequently infected with either XMRV_VP62 _or XMRV_22Rv1 _(Figure 1). The resultant stocks (XMRV+LtatSN) were mixtures of native XMRV and XMRV-pseudotyped virions [LtatSN(XMRV)] in which LtatSN RNA was packaged together with XMRV Gag, Pol and Env proteins; only the LtatSN(XMRV) fraction was detected in subsequent culture steps. To quantify drug susceptibility, MAGIC-5A cultures were treated with varying concentrations of NRTIs, NNRTIs, or integrase inhibitors, and infected with XMRV_VP62_+LtatSN or XMRV_22Rv1_+LtatSN (Figure 1). Entry of XMRV occurs through the interaction of the virus with xenotropic and polytropic retrovirus receptor 1 (XPR1), which is endogenously expressed in HeLa cell lines [28]. XMRV+LtatSN infection of MAGIC-5A cells induced the expression of β-galactosidase (β-gal) via Tat-mediated transactivation of an upstream HIV-1 LTR, thereby enabling us to quantify the dose-dependent reduction of β-gal^+ ^foci in infected indicator cell cultures. For assays of protease inhibitor (PI) susceptibility, XMRV-infected HTX/LtatSN cells were seeded in microtiter plates and immediately treated with PIs. Following a two-day incubation period, samples from the PI-treated HTX cultures were transferred to MAGIC-5A cells for FFU determination. MAGIC-5A cells also express receptors and coreceptors for HIV-1 entry (CD4, CXCR4 and CCR5; Figure 1), and thus, we were able to perform side-by-side comparisons of the drug susceptibilities of XMRV and HIV-1 in the same host cell type. In both cases, viral replication was limited to a single cycle of infection.

### XMRV is susceptible to a specific subset of NRTIs

We initially measured the susceptibility of XMRV to each of seven different NRTIs that are FDA-approved for treating HIV-1 infection. AZT showed the most potent anti-XMRV activity of all the nucleoside analogs tested (Table 1); EC_50 _values for XMRV_VP62_+LtatSN, XMRV_22Rv1_+LtatSN and HIV-1_NL4-3 _were similar for AZT, indicating that these viruses are comparably susceptible to the analog. These results agree with a previous comparison of the AZT sensitivity of HIV-1 and XMRV using a reporter virus-based assay [22]. We also found that, relative to HIV-1_NL4-3_, XMRV_VP62_+LtatSN and XMRV_22Rv1_+LtatSN were fully sensitive to tenofovir (the active form of TDF), as the observed EC_50 _values were not significantly different between these three viruses (Table 1). In contrast, XMRV was 13-34-fold resistant to ddI, d4T and abacavir relative to HIV-1_NL4-3_. Higher levels of resistance were observed for 3TC and FTC, which failed to inhibit XMRV infection at doses that were 100-fold greater than the corresponding EC_50_s for HIV-1_NL4-3_.

**Table 1:** Susceptibility of XMRV and HIV-1 to reverse transcriptase inhibitors.

**Inhibitor class**^**b**^	**Inhibitor**^**c**^	**EC**_**50 **_**(μM)**^**a**^		
		
		**HIV-1**_**NL4-3**_	**XMRV**_**VP62**_**+LtatSN**^**d**^	**XMRV**_**22Rv1**_**+LtatSN**^**d**^
NRTI	AZT	0.10 ± 0.05	0.12 ± 0.03 (1)	0.06 ± 0.02 (1)
	AZddG	0.71 ± 0.01	1.1 ± 0.1 (2)	**2.7 ± 0.7 (4)**
	AZddA	2.0 ± 0.9	1.6 ± 0.4 (1)	3.2 ± 1.2 (2)
	tenofovir	3.5 ± 0.9	5.8 ± 3.2 (2)	5.3 ± 3.8 (2)
	adefovir	14 ± 2	9.5 ± 3.7 (1)	7.0 ± 0.8 (0.5)
	D4T	0.99 ± 0.53	**34 ± 22 (34)**	**13 ± 1 (13)**
	ddI	1.79 ± 0.04	**43 ± 23 (24)**	**43 ± 12 (24)**
	abacavir	3.6 ± 1.9	**94 ± 54 (26)**	**66 ± 39 (18)**
	3TC	0.35 ± 0.07	**> 40 (> 100)**	**> 40 (> 100)**
	FTC	0.059 ± 0.041	**> 40 (> 100)**	**> 40 (> 100)**
NNRTI	efavirenz	0.005 ± 0.002	**> 1 (> 200)**	**> 1 (> 200)**
	nevirapine	0.22 ± 0.07	**> 4 (> 18)**	**> 4 (> 18)**
PP_i _analog	PFA	126 ± 93	**> 400 (> 3)**	**> 400 (> 3)**

^a ^EC_50 _values were measured in MAGIC-5A cells as described in Methods and are the means ± standard deviation from two or more independent experiments. Numbers in parentheses indicate the fold change in EC_50 _relative to HIV-1_NL4-3_. Values shown in bold are significantly different from the corresponding values for HIV-1_NL4-3 _(p < 0.05, ANOVA with Tukey's multiple comparison test).

^b ^NRTI, nucleoside reverse transcriptase inhibitor. NNRTI, non-nucleoside reverse transcriptase inhibitor. PP_i _analog, pyrophosphate analog.

^c ^See Abbreviations for drug names.

^d ^XMRV-pseudotyped LtatSN virus. See text for details.

To further characterize the nucleoside analog susceptibility of XMRV, we determined the antiviral activities of additional NRTIs that are active against HIV-1 and other retroviruses, but that are not currently approved for treating HIV-1 infection. AZddA and AZddG contain an azido group at the 3' position of the ribosyl sugar, and thus, are structurally related to AZT. AZddA and AZddG have been shown to inhibit HIV-1 replication in culture, and the 5'-triphosphate forms of these analogs inhibit the DNA polymerase activity of HIV-1 RT in cell-free assays [29]. EC_50 _values for the inhibition of XMRV and HIV-1 by AZddA and AZddG were comparable, although the EC_50 _for XMRV_22Rv1_+LtatSN with AZddG was fourfold greater than that of HIV-1_NL4-3 _(Table 1). Importantly, the concentrations of AZddA, AZddG and AZT required to inhibit XMRV infection were at least 100-fold lower than the 50% cytotoxic concentrations (CC_50 _values) of these analogs in HeLa-CD4 cell cultures (> 270 μM for all three inhibitors; [29]). We also measured the anti-XMRV activity of adefovir, an acyclic nucleoside phosphonate that is used in prodrug form (adefovir dipivoxil) to treat hepatitis B virus infection. EC_50 _measurements for the activity of adefovir against XMRV_VP62_+LtatSN, XMRV_22Rv1_+LtatSN and HIV-1_NL4-3 _varied by a factor of twofold or less; these differences were not statistically significant (Table 1).

Taken together, these data show that XMRV is sensitive to AZT, AZddA, AZddG, tenofovir and adefovir at doses that are comparable to those required to inhibit HIV-1 replication. At the highest concentrations of the drugs used in our assays (10 μM for AZT, 40 μM for AZddA and AZddG and 100 μM for adefovir and tenofovir), the mean numbers of cells in the fixed and stained cultures were 80-100% of untreated controls, indicating that the EC_50 _values obtained for these analogs were not influenced by drug-mediated cytotoxicity.

### XMRV is resistant to NNRTIs and to the pyrophosphate analog foscarnet

Nevirapine, efavirenz and other NNRTIs inhibit HIV-1 RT by binding to a small hydrophobic pocket located near the polymerase active site [30]. Although wild-type strains of HIV-1 Group M are sensitive to NNRTIs, HIV type 2 (HIV-2), simian immunodeficiency virus and many Group O isolates of HIV-1 are intrinsically resistant to this drug class. Consistent with the relatively narrow spectrum of NNRTI-mediated antiviral activity, both strains of XMRV were >18-fold and >200-fold resistant to nevirapine and efavirenz, respectively, relative to HIV-1_NL4-3 _(Table 1). In contrast, the pyrophosphate analog foscarnet (PFA) is active against many DNA viruses and retroviruses including HIV-1 and -2, Rauscher MLV, Moloney MLV, hepatitis B virus, cytomegalovirus and herpes simplex virus [31]. Despite this broad spectrum of antiviral activity, XMRV_VP62_+LtatSN and XMRV_22Rv1_+LtatSN were resistant to PFA (Table 1). Concentrations of PFA as high as 400 μM had no effect on XMRV infection; increasing the drug level to 900 μM produced visible cytotoxic effects in MAGIC-5A indicator cell cultures (data not shown).

### XMRV is intrinsically resistant to PIs but is sensitive to integrase inhibitors

**Figure 2:**
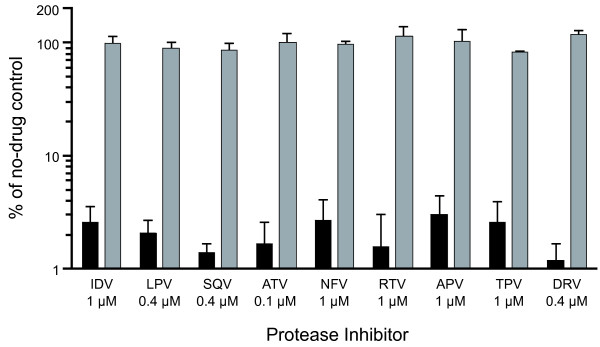
**Intrinsic resistance of XMRV to protease inhibitors (PIs)**. For XMRV_VP62_+LtatSN (shaded bars), HTX/LtatSN cells were infected with virus derived from the pVP62 clone, seeded into microtiter plates, and immediately treated with the indicated doses of PIs. For HIV-1_NL4-3 _(solid bars), 293T/17 cells were seeded into microtiter plates, transfected with plasmid DNA encoding the full-length NL4-3 molecular clone, and treated with the indicated concentrations of each PI. The same PI stocks were used to treat both sets of virus-producing cultures. Supernatants from PI-treated HTX and 293T/17 cultures were then diluted and plated onto MAGIC-5A indicator cells to quantify infectious particles. Bars represent the percentage of β-gal^+ ^FFU in supernatants from the PI-treated cultures, relative to untreated controls, and are the means ± standard deviations from two independent experiments with two or more determinations of FFU per drug treatment per experiment. See List of Abbreviations for drug names.

To identify antivirals that inhibit XMRV targets other than RT, we assessed the ability of nine different HIV-1 PIs to block the production of newly-formed, infectious XMRV_VP62_+LtatSN in chronically-infected HTX cultures. In these experiments, we screened each PI for anti-XMRV activity using a single drug concentration that was approximately equal to the EC_95 _for HIV-1_NL4-3_, as determined in our concurrent studies of HIV-1 and HIV-2 (range = 0.1-1 μM; see Methods section for details). As seen in our previous assays, these PI doses reduced the infectious titer of HIV-1_NL4-3 _in pNL4-3-transfected 293T/17 cultures by 94% or greater, relative to untreated controls (Figure 2). In contrast, each of the nine PI treatments had no detectable effect on the infectious titer of XMRV_VP62_+LtatSN, indicating that XMRV_VP62 _is intrinsically resistant to this inhibitor class. These results are consistent with a recent report showing that XMRV is relatively insensitive to PIs (EC_50 _values ≥34 μM) in cultures of immortalized human breast cancer cells [23].

We also examined the susceptibility of XMRV to two different inhibitors of HIV-1 integrase strand-transfer activity: raltegravir and elvitegravir. Of the 24 antiretroviral drugs tested in our analysis, raltegravir was the most potent inhibitor of XMRV infection. XMRV and HIV-1 exhibited comparable sensitivity to raltegravir, as the EC_50 _values for XMRV_VP62_+LtatSN and XMRV_22Rv1_+LtatSN were similar to that of HIV-1_NL4-3 _(Table 2). Elvitegravir also inhibited XMRV infection in our indicator cell assays, but higher doses of the drug were required to observe this activity. EC_50 _measurements for XMRV_VP62_+LtatSN and XMRV_22Rv1_+LtatSN were 71- and 40-fold greater for elvitegravir relative to raltegravir and 79- and 46-fold higher than the EC_50 _for elvitegravir-mediated inhibition of HIV-1_NL4-3_, respectively (Table 2). Although these data show that elvitegravir is less potent than raltegravir against XMRV, we note that elvitegravir inhibited the virus at concentrations in the nanomolar range, and thus, was comparable to AZT with respect to anti-XMRV activity (Tables 1 and 2). For both raltegravir and elvitegravir, no statistically-significant declines in mean target cell number were observed at the highest doses of drugs tested (10 μM; p > 0.05, Student's two-sided t-test). This result agrees with previously-published CC_50 _values for raltegravir and elvitegravir in PBMC (> 100 μM and 40 μM, respectively; [23,32]) and excludes cytotoxicity as a potential confounder in our measurements of integrase inhibitor susceptibility.

**Table 2:** Susceptibility of XMRV and HIV-1 to integrase inhibitors.

	**EC**_**50 **_**(nM)**^**a**^		
	
Inhibitor	**HIV-1**_**NL4-3**_	**XMRV**_**VP62**_**+LtatSN**^**b**^	**XMRV**_**22Rv1**_**+LtatSN**^**b**^
raltegravir	3.7 ± 2.1	2.1 ± 1.1 (1)	2.2 ± 1.1 (1)
elvitegravir	1.9 ± 0.7	**150 ± 115 (79)**	**87 ± 29 (46)**

^a ^EC_50 _values were measured in MAGIC-5A cells as described in Methods and are the means ± standard deviation from two or more independent experiments. Numbers in parentheses indicate the fold change in EC_50 _relative to HIV-1_NL4-3_. Values shown in bold are significantly different from the corresponding values for HIV-1_NL4-3 _(p < 0.05, ANOVA with Tukey's multiple comparison test).

^b ^XMRV-pseudotyped LtatSN virus. See text for details.

## Discussion

In this study, we used a novel marker transfer assay to directly compare the susceptibility of XMRV and HIV-1 to a panel of antiretroviral drugs in the same host cell type. Our experimental approach and findings differ from previous studies of XMRV in several important ways. With regard to NRTIs, the initial report by Sakuma *et al. *[21] suggested that XMRV is sensitive to AZT but resistant to 3TC, d4T and tenofovir. Importantly, the single dose of tenofovir used in their experiments (30 nM) was substantially lower than the EC_50 _observed in our assays (~5 μM; Table 1), leading the authors to conclude that XMRV was resistant to the drug. Our analysis shows that tenofovir is equally potent against XMRV and HIV-1 in culture (Table 1). A subsequent study by Singh *et al. *[23] used differing cell types to compare XMRV and HIV-1, and as a result, differences in the intrinsic NRTI susceptibilities of the two viruses could not be resolved from host cell-specific differences in NRTI activity. In fact, careful inspection of their data suggests that XMRV is relatively resistant to AZT, tenofovir and TDF (a prodrug of tenofovir), as the EC_50 _values for these analogs were 15-94-fold higher for XMRV compared to HIV-1. Our data are more congruent with the findings of Paprotka *et al. *[22], who showed that XMRV and HIV-1 are comparably sensitive to AZT and TDF in prostate cancer cells. We extend these observations by demonstrating that, in addition to AZT and tenofovir, the NRTIs AZddA, AZddG and adefovir are equally active against XMRV and HIV-1 (Table 1). Taken together, our analysis resolves disparities among earlier reports of XMRV drug susceptibility and illustrates that XMRV is sensitive to a broader range of NRTIs than was previously appreciated.

Overall, the patterns of drug susceptibility observed in our analysis of XMRV are similar to those seen in previous studies of Moloney MLV (MoMLV). MoMLV is sensitive to AZT, adefovir and tenofovir, but is relatively resistant to ddI, D4T, 3TC, abacavir and PFA [33-36]. In addition, purified MoMLV protease is highly resistant to PIs [37], whereas both raltegravir and elvitegravir have been shown to inhibit MoMLV replication in culture [38,39]. In agreement with our findings for XMRV (Table 2), MoMLV is moderately resistant to elvitegravir, as evidenced by a 7-fold greater EC_50 _for the drug relative to HIV-1 [39]. These concurrent drug sensitivity patterns are consistent with the high degree of amino acid sequence similarity shared between XMRV and MoMLV, which are 99% identical in the protease and RT polymerase domain and 90% identical in the integrase catalytic core domain (CCD).

**Figure 3:**
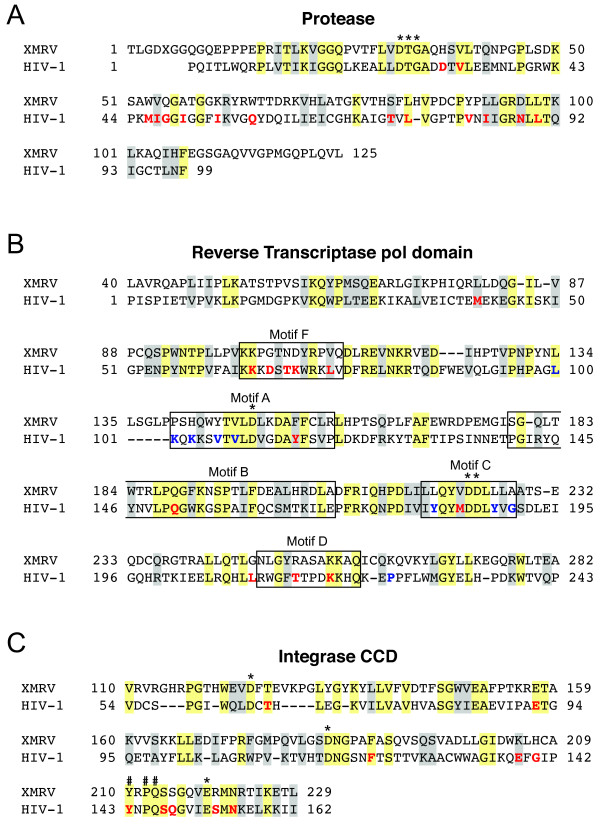
**Alignment of Pro and Pol amino acid sequences for XMRV and HIV-1**. Alignments are shown for the protease (panel A), amino-terminal RT (panel B) and integrase catalytic core domain (CCD) sequences (panel C) of XMRV_VP62 _and HIV-1_NL4-3 _([GenBank: NC_007815.1] and [GenBank: M19921], respectively). Numbering for XMRV_VP62 _is based on assigned amino acid numbers for the corresponding MoMLV peptides [GenBank: AF033811]. Alignments were generated using EMBOSS [62] with the following settings: gap-open = 10, gap extend = 0.5, algorithm = needle (global), scoring matrix = BLOSUM62. Amino acid identities between XMRV_VP62 _and HIV-1_NL4-3 _are shown with yellow boxes, conserved amino acid residues (BLOSUM62 score ≥1) are shown with grey boxes, and alignment gaps with are indicated with a dash (-). Catalytic active site residues are indicated with an asterisk (*). For RT, the initial EMBOSS alignment was manually adjusted to conform to a recent structural alignment of MoMLV and HIV-1 RTs [63]. Boundary boxes for conserved polymerase motifs A-D are shown as previously assigned [64]. Boundaries for motif F are shown as identified in alignments of viral RNA-dependent RNA polymerases [65]. The X at position five of XMRV protease indicates the location of a termination codon that, in MLV, is suppressed during translation of Gag-Pol-encoding RNA. Sites involved in antiretroviral drug resistance in HIV-1, as tabulated by the International AIDS Society-USA (for protease and RT) [40] or in the Stanford University HIV Drug Resistance Database (for integrase) [66] are indicated in bold, colored letters. The locations of primary PI, NRTI, and NNRTI resistance mutations, as well as changes associated with resistance to the integrase inhibitors raltegravir and elvitegravir, are shown in red. Sites involved in NNRTI resistance are shown in blue. Pound signs (#) indicate amino acid residues believed to be important for the positioning of strand transfer inhibitors, based on a recent structural analysis of prototype foamy virus integrase [51].

To gain further insights into the molecular basis of antiretroviral drug resistance in XMRV, we constructed amino acid alignments of the inferred XMRV_VP62 _and HIV-1_NL4-3 _sequences for the entire protease enzyme, the portion of RT spanning the conserved polymerase motifs, and the integrase CCD (Figure 3). Within these three regions, XMRV and HIV-1 share 27-31% amino acid identity and 18-21% amino acid similarity. Importantly, the XMRV and HIV-1 sequences differ at several sites that are critical for antiretroviral drug resistance. XMRV protease contains three residues (V54, S81, and L92) that correspond to PI resistance-conferring replacements in HIV-1 (I47V, T74 S, and I84L, respectively) (Figure 3A) [40]. XMRV also contains several amino acid residues in the RT polymerase domain that, in HIV-1, result in NNRTI resistance (K101P, K103 H, Y181L, Y188L, and G190A) and dideoxynucleoside analog resistance (T69N, L74V, Y115F) (Figure 3B) [40,41]. These sites likely contribute to intrinsic drug resistance in XMRV. In addition, XMRV integrase contains a serine at the position corresponding to Q148 in HIV-1 (Figure 3C), which is known to be critical for integrase inhibitor resistance in HIV-1 [42]. This amino acid difference may contribute to moderate elvitegravir resistance in XMRV (Table 2).

As observed in previous studies of MoMLV RT [43,44], XMRV was highly resistant to the L-pseudosugar nucleoside analogs 3TC and FTC (Table 1). Both MoMLV and XMRV RT encode a valine at the second position of the conserved YXDD sequence of polymerase motif C, whereas the corresponding residue in HIV-1 RT is methionine 184 (Figure 3B). Although the M184V replacement confers high-level resistance to 3TC and FTC in HIV-1 [45], mutants of MoMLV that harbor the reciprocal change in the YXDD sequence (V223M) remain highly resistant to 3TC [43,44]. It is therefore likely that amino acid sites outside the YXDD sequence of RT contribute to intrinsic 3TC/FTC resistance in XMRV.

In HIV-1 RT, specific substitutions at positions 41, 67, 70, 210, 215 and 219 (commonly known as thymidine analog mutations or TAMs) confer AZT resistance by enhancing RT-catalyzed excision of AZT-5'-monophosphate from the nascent DNA strand [46]. Although the sequences of XMRV and HIV-1 differ at five of the six TAM sites in RT (Figure 3B), these residues are unlikely to influence AZT susceptibility in XMRV, as the excision activity of MoMLV RT is orders of magnitude lower than that of the HIV-1 enzyme [47]. Indeed, we observed that XMRV and HIV-1 were comparably sensitive to AZT as well as two other NRTIs containing a 3'-azido modification (AZddA and AZddG; Table 1). Based on previous studies of HIV-1 and MoMLV [29,48,49], we expect that XMRV RT can utilize the 5'-triphosphate forms of these analogs as alternative nucleotide substrates, resulting in chain termination of DNA synthesis. Additional biochemical analyses are required to characterize the nucleotide selectivity and excision activity of XMRV RT.

Two recently-published reports have shown that the integrase inhibitor raltegravir inhibits XMRV replication in culture at nanomolar concentrations of the drug [22,23]. Our results confirm these findings and demonstrate that elvitegravir is also active against XMRV, although the concentrations of elvitegravir needed to inhibit XMRV infection were higher than those required for raltegravir (Table 2). A third integrase inhibitor, L-870812, has also been reported to exert moderate antiviral activity against XMRV in culture, with an EC_50 _32-fold greater than that of raltegravir [23]. Although raltegravir, elvitegravir and L-870812 are structurally divergent, these three inhibitors share a common pharmacophore that binds the active site metal ions essential for integrase strand transfer catalysis [50]. Recent crystallographic studies have identified three amino acid residues that are believed to influence the positioning of strand transfer inhibitors in the integrase active site [51], and based on our alignment of the CCD, these residues are conserved in the XMRV and HIV-1 integrase sequences (Figure 3C). Taken together, these data suggest that the strand transfer inhibitor-binding sites of XMRV and HIV-1 integrase share a similar overall topology despite numerous amino acid differences in the CCD.

**Figure 4:**
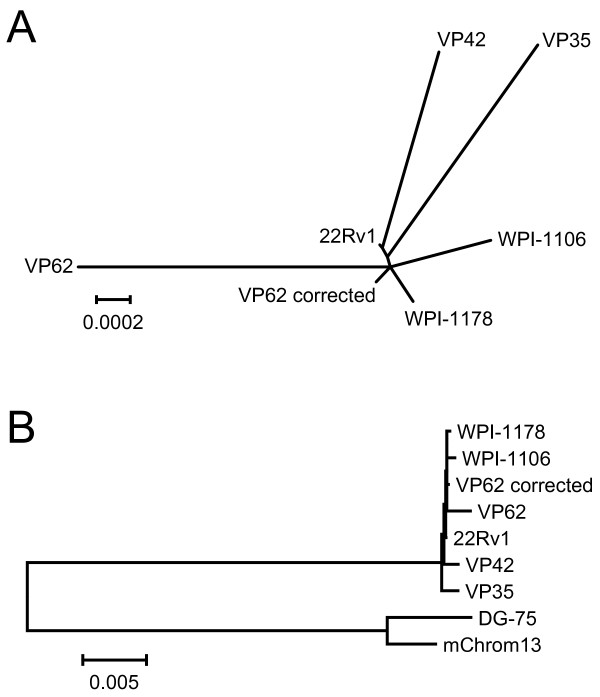
**Phylogenetic analysis of XMRV**. All full-length XMRV sequences available in GenBank (accessed on April 28, 2010) were aligned using ClustalW. Unrooted (panel A) and rooted (panel B) phylogenetic trees were generated using the neighbor-joining algorithm of MEGA 4.0 [67] with default settings. Scale bars indicate evolutionary distance in base substitutions per site (*i.e*., the distance shown in panel A equals 2 substitutions per 10,000 bases). Note that after the original sequencing of XMRV strains VP62, VP42 and VP35 [3], strain VP62 was resequenced ("VP62 corrected"; [11]). The resulting sequence reveals a closer similarity between VP62 and other XMRV strains and suggests that the branch lengths of VP35 and VP42 are also likely overestimated due to PCR or sequencing errors. mChrom13 indicates an endogenous MLV sequence located on *Mus musculus *chromosome 13 [GenBank: CT030655.7], and is the most closely related non-XMRV sequence found by BLAST search of GenBank using the XMRV_22Rv1 _sequence. DG-75 indicates DG-75 MLV [GenBank: AF221065].

We used two independent sources of XMRV for our studies: one derived from the infectious molecular clone VP62 [11] and the other from 22Rv1 prostate carcinoma cells [7]. Our rationale for this choice was that the VP62 clone might encode alterations that influence drug susceptibility, whereas 22Rv1 cells harbor at least 10 proviral copies of XMRV, presumably providing a more diverse sample of the virus. However, a recent analysis of XMRV sequences from 22Rv1 cells revealed that the proviruses are nearly identical to each other and to the VP62 molecular clone [22]. There are only two nucleotide differences between the consensus XMRV_22Rv1 _and XMRV_VP62 _sequences ([GenBank: FN6900043] and [GenBank: EF185282], respectively); these result in single amino acid changes in Gag and Env, whereas the Pro and Pol proteins are identical. Thus, the key proteins targeted by the antiretroviral drugs tested in our study are identical in XMRV_22Rv1 _and XMRV_VP62_. This identity is reflected in the similar EC_50 _values obtained for these two viruses (Tables 1 and 2). Strikingly, all six of the full-length XMRV sequences currently available in GenBank show a high degree of nucleotide identity (Figure 4). Although the lack of variation reported in XMRV is difficult to reconcile with the known mutation rates of MoMLV and other retroviruses, collectively, these sequencing results suggest that the drugs that are active against XMRV_22Rv1 _and XMRV_VP62 _should be similarly active against other XMRV strains.

## Conclusions

Our analysis demonstrates that XMRV is sensitive to a broader range of NRTIs than was previously appreciated; these include analogs that are used in the clinical treatment of HIV-1 infection (AZT and tenofovir) as well as other structurally-related NRTIs (AZddA, AZddG and adefovir). We observed a distinct pattern of NRTI sensitivity in XMRV that correlates with the structure of the pseudosugar moiety; while XMRV is sensitive to 3'-azido nucleoside analogs and acyclic nucleoside phosphonates, the virus is moderately resistant to dideoxynucleosides and highly resistant to L-form thiacytidine NRTIs. Importantly, this pattern suggests that other 3'-azido or acyclic nucleoside analogs might also exhibit anti-XMRV activity. In addition, our data show that elvitegravir blocks XMRV infection with a degree of potency similar to that of AZT. This finding expands the number of integrase inhibitors with known activity against XMRV *in vitro*.

While our use of the same target cell type for XMRV and HIV-1 provides an important reference point for characterizing XMRV drug susceptibility, we note that the two viruses utilize different receptors for entry and are therefore likely to infect differing host cell types *in vivo*. Ultimately, the clinical utility of antiretrovirals for XMRV will depend on drug distribution and metabolism at anatomic sites of XMRV replication, the degree to which antiretrovirals reduce XMRV viral load, and whether reductions in viral load slow pathogenesis. In the event that XMRV is shown to be the causative agent of human disease, our data identify candidate drugs for clinical studies of antiretroviral therapy in XMRV-infected patients.

## Methods

### Inhibitors

AZT (generic name: zidovudine; 3'-azido-3'-deoxythymidine), ddI (didanosine; 2',3'-dideoxyinosine), D4T (stavudine; 2',3'-didehydro-3'-deoxythymidine) and PFA (foscarnet; phosphonoformic acid) were obtained commercially (Sigma-Aldrich), as were adefovir ((*R*)-9-(2-phosphonylmethoxyethyl)adenine), tenofovir ((*R*)-9-(2-phosphonylmethoxypropyl)adenine) and abacavir ((1*S*,4*R*)-4-[2-amino-6-(cyclopropylamino)-9*H*-purin-9-yl]-2-cyclopentene-1-methanol) (Moravek Biochemicals), AZddA (3'-azido-2',3'-dideoxyadeonsine) and AZddG (3'-azido-2',3'-dideoxyguanosine) (Berry and Associates), and elvitegravir (Selleck Chemicals). Nevirapine and efavirenz were a gift from Koronis Pharmaceuticals (Seattle, Washington). 3TC (lamivudine; (-)-β-L-2',3'-dideoxy-3'-thiacytidine) and FTC (emtricitabine; (-)-β-L-2',3'-dideoxy-5-fluoro-3'-thiacytidine) were kindly provided by Raymond Schinazi (Emory University) or were purchased from Moravek. All HIV-1 PIs used in this study, as well as the integrase inhibitor raltegravir, were obtained from the National Institutes of Health AIDS Reference Reagent Program.

### Cell culture and virus production

HTX cells are a pseudodiploid subclone of HT-1080 human fibrosarcoma cells [52]. The LtatSN vector was created by inserting the *tat *coding region of HIV strain SF2 into the retroviral expression vector LXSN [53]. HTX/LtatSN cells were generated by infecting HTX cells with helper-virus free LtatSN virus that was produced in PA317 amphotropic packaging cells [54] and then treating the cells with G418 (geneticin) to select for the presence of the vector. 22Rv1 cells [27] and 293T/17 cells [55] were obtained from the American Type Culture Collection. MAGIC-5A indicator cells (CD4^+^/CCR5^+ ^HeLa cells that express β-galactosidase (β-gal) under the control of an HIV-1 LTR promoter) [26] were a kind gift from Dr. Michael Emerman (Fred Hutchinson Cancer Research Center). Cell lines were cultured in Dulbecco's Modified Eagle's Medium (DMEM) supplemented with 10% fetal bovine serum.

XMRV-pseudotyped LtatSN virus (XMRV+LtatSN) was generated by infecting HTX/LtatSN cells with virus produced from the VP62 molecular clone of XMRV (a kind gift from Robert Silverman, Cleveland Clinic) [11] or with virus harvested from XMRV-infected 22Rv1 cells [7]. HIV-1_NL4-3 _was produced using the full-length pNL4-3 HIV-1 plasmid molecular clone [56]. Plasmid DNA was isolated from pNL4-3-transformed *E. coli *JM109 using an Endo-Free™ maxiprep kit (Qiagen) and introduced into cultured 293T/17 cells via chloroquine-mediated transfection as previously described [57]. XMRV_VP62_+LtatSN, XMRV_22Rv1_+LtatSN and HIV-1_NL4-3 _stocks were harvested from confluent monolayers of producer cells, passed through 0.45-micron filters (XMRV+LtatSN) or centrifuged at 500 × *g *for 10 min at room temperature (HIV-1_NL4-3_) to remove host cells, and frozen in multiple aliquots at -70°C. Titers of the resultant stocks were 7.3 × 10^5^, 1.2 × 10^5^, and 3.0 × 10^6 ^MAGIC-5A focus forming units (FFU)/ml for XMRV_VP62_+LtatSN, XMRV_22Rv1_+LtatSN and HIV-1_NL4-3_, respectively.

### Drug Susceptibility Assays-RT and Integrase Inhibitors

To compare the susceptibilities of XMRV and HIV-1 to NRTIs, NNRTIs and PFA, MAGIC-5A cells were seeded into 48-well plates at 1.5 × 10^4 ^cells/well. After 20-22 h of incubation, the cultures were dosed with varying drug concentrations and returned to the incubator for an additional 2.5 h. Immediately before infection, virus stocks were diluted to 3,000 FFU/ml in complete DMEM supplemented with 20 μg/ml diethylaminoethyl (DEAE) dextran. Supernatants from the drug-treated MAGIC-5A cultures were then aspirated and replaced with 100 μl of each diluted virus stock/well. To maintain drug pressure, a second dose of inhibitor was added to the inocula (at the same concentration as the first dose), and the plates were returned to the incubator for 2.5 h. After this time, an additional 300 μl of complete DMEM was added, a third dose of drug was added, and incubation was continued for 40 h. Individual dose-response experiments for each virus strain involved 2-3 solvent-only control cultures plus 2-3 cultures for each of seven different drug concentrations.

To score β-gal-positive (β-gal^+^) foci, 100 μl of fixative solution [1% formaldehyde, 0.2% glutaraldehyde in 1× phosphate-buffered saline (PBS)] was added to each culture well, and the plates were incubated at 37°C for 10 min. After washing the fixed monolayers twice with 100 μl of PBS, 100 μl of staining solution [4 mM potassium ferrocyanide, 4 mM potassium ferricyanide, 2 mM MgCl_2 _and 0.4 mg/ml 5-bromo-4-chloro-3-indolyl-β-D-galactopyranoside (X-gal) in PBS] was added to each well, and the plates were placed in the incubator for 1 h. The cultures were then aspirated to remove the X-gal staining solution, rinsed with 100 μl of PBS per well, aspirated again and stored in 200 μl of PBS per well. Foci (individual β-gal^+ ^cells plus groups of 2-8 contiguous β-gal^+ ^cells) were counted using a CTL Immunospot Analyzer (Cellular Technology Ltd.) or were manually counted by light microscopy. Untreated control cultures typically contained 200-500 foci per well.

To quantify viral susceptibility to integrase inhibitors, we adopted our MAGIC-5A-based assay to a 96-well format and used an expanded range of drug concentrations. These changes were necessitated by the shallow slopes observed in dose-response plots with raltegravir and elvitegravir relative to inhibitors from other drug classes [58]. Culture conditions and times of drug addition were identical to those used for the RT inhibitor assays, except that each culture well was seeded with 5 × 10^3 ^MAGIC-5A cells in 100 μl of medium, was infected with 200 FFU of virus in 50 μl of dextran-containing medium, and received an additional 150 μl of complete medium following the 2.5 h incubation period. Fixing and X-gal-staining steps were performed with one half of the volumes of solutions used in RT inhibitor assays, and β-gal^+ ^foci were counted using the CTL Immunospot Analyzer.

Drug concentrations that inhibited focus formation by 50% (EC_50 _values) were calculated from dose-response plots by sigmoidal regression analysis (GraphPad Software). EC_50 _measurements for HIV-1_NL4-3 _were comparable to the values obtained in other single-cycle drug sensitivity assays [26,59,60].

Potential drug-mediated cytotoxicity was assessed by comparing the number of cells in untreated control cultures to those in cultures that received the maximal dosage of drug used in our assays. Fixed cells were stained by exposing the MAGIC-5A monolayers to 10 μg/ml ethidium bromide in PBS for 5 min, then de-staining for 5 min in deionized water. Cell nuclei were visualized by fluorescence microscopy using a Texas red filter set (560 nm excitation, 645 nm emission). Images were acquired from 3-4 culture wells for each drug treatment and corresponding no-drug controls, and nuclei were enumerated using ImageJ software [61].

### Drug Susceptibility Assays-Protease Inhibitors

To measure PI susceptibility, cultured cells that were producing either HIV-1 or XMRV were treated with varying doses of PIs, and the numbers of infectious virions released by each drug-treated or no-drug control culture were quantified in MAGIC-5A indicator cells. For HIV-1_NL4-3_, 293T/17 cells grown in 75 cm^2 ^flasks were digested with trypsin, seeded into 48-well plates at 6 × 10^4 ^cells/well, and placed in an incubator. The following day (20-24 h), CaPO_4_-DNA co-precipitates were prepared by mixing 5 μg of HIV-1_NL4-3 _plasmid DNA with 900 μl of 0.2 M CaCl_2_, adding the solution dropwise with mixing into 900 μl of 2× Hepes-buffered saline, and then incubating the suspension at room temperature for 10 min. During this time, chloroquine was added to each 293T/17 culture well to a final concentration of 50 μM. Co-precipitate suspensions were then mixed by pipetting and added directly to the chloroquine-treated cultures (20 μl/well), and the plates were placed in the incubator for 10-12 h. Following this incubation period, the supernatants were aspirated and replaced with 400 μl of fresh medium per well, and PIs were added to the culture wells. The plates were then returned to the incubator for 30-35 h. Supernatants (20 μl) from the transfected 293T/17 cultures were removed without disturbing the cell monolayer and diluted 1:10, 1:100 and 1:1,000 in complete medium supplemented with 20 μg/ml DEAE dextran. Infectious titers in the diluted supernatants were measured in MAGIC-5A cells as described above, except that inhibitors were omitted from this phase of the assay.

For PI susceptibility assays with XMRV, HTX/LtatSN cells that were infected with XMRV_VP62 _were trypsinized, rinsed twice with 1× PBS, resuspended in complete medium and seeded into 48-well plates at approximately 5 × 10^4 ^cells/well. The cultures were then immediately treated with PIs as described above for HIV-1_NL4-3_. Following a 40-h incubation period, 180 μl of culture supernatant was harvested from each well, and DEAE-dextran was added to the samples to a final concentration of 20 μg/ml. The supernatants were diluted 1:4 and 1:16 in medium containing 20 μg/ml DEAE dextran, and 100 μl each of the undiluted, 1:4- and 1:16-diluted samples were transferred to MAGIC-5A cultures for FFU determination as described above.

## Abbreviations

XMRV: xenotropic murine leukemia virus-related virus; HIV-1: human immunodeficiency virus type 1; RT: reverse transcriptase; NRTI: nucleoside reverse transcriptase inhibitor; NNRTI: non-nucleoside reverse transcriptase inhibitor; PI: protease inhibitor; AZT: generic name-zidovudine, 3'-azido-3'-deoxythymidine; AZddA: 3'-azido-2',3'-dideoxyadenosine; AZddG: 3'-azido-2',3'-dideoxyguanosine; adefovir: (*R*)-9-(2-phosphonylmethoxyethyl)adenine; tenofovir: (*R*)-9-(2-phosphonylmethoxypropyl)adenine; ddI: didanosine, 2',3'-dideoxyinosine; TDF: tenofovir disoproxil fumarate; d4T: stavudine, 2',3'-didehydro-3'-deoxythymidine; abacavir: (1*S*,4*R*)-4-[2-amino-6-(cyclopropylamino)-9*H*-purin-9-yl]-2-cyclopentene-1-methanol; 3TC: lamivudine, (-)-β-L-2',3'-dideoxy-3'-thiacytidine; FTC: emtricitabine, (-)-β-L-2',3'-dideoxy-5-fluoro-3'-thiacytidine; PFA: foscarnet, phosphonoformic acid; IDV: indinavir; LPV: lopinavir; SQV: saquinavir; ATV: atazanavir; NFV: nelfinavir; RTV: ritonavir; APV: amprenavir; TPV: tipranavir; DRV: darunavir; FFU: focus-forming units; EC_50_: the concentration of drug required to inhibit infection by 50%; ANOVA: analysis of variance; FDA: United States Food and Drug Administration.

## Competing interests

The authors declare that they have no competing interests.

## Authors' contributions

RAS contributed to the experimental design, prepared essential reagents, acquired and analyzed the drug susceptibility data, and drafted the manuscript. ADM contributed to the experimental design, prepared essential reagents, performed the phylogenetic analysis of XMRV sequences, assisted in data acquisition and interpretation, and helped prepare the manuscript. GSG performed amino acid alignments of XMRV and HIV-1 sequences, assisted with data interpretation, contributed to the phylogenetic analysis of XMRV sequences, and helped prepare the manuscript. All authors read and approved the final manuscript.
